# Supplementary material for: Detection of Porcine Circovirus Type 3 in Free-Ranging Wild Boars and Ticks in Jiangsu Province, China
Source: Viruses. 2025 Jul 28;17(8):1049. doi: 10.3390/v17081049 (PMC12390564; doi:10.3390/v17081049)
Supplement: Supplementary file 1 [file viruses-17-01049-s001.zip › Figure S1.pdf]

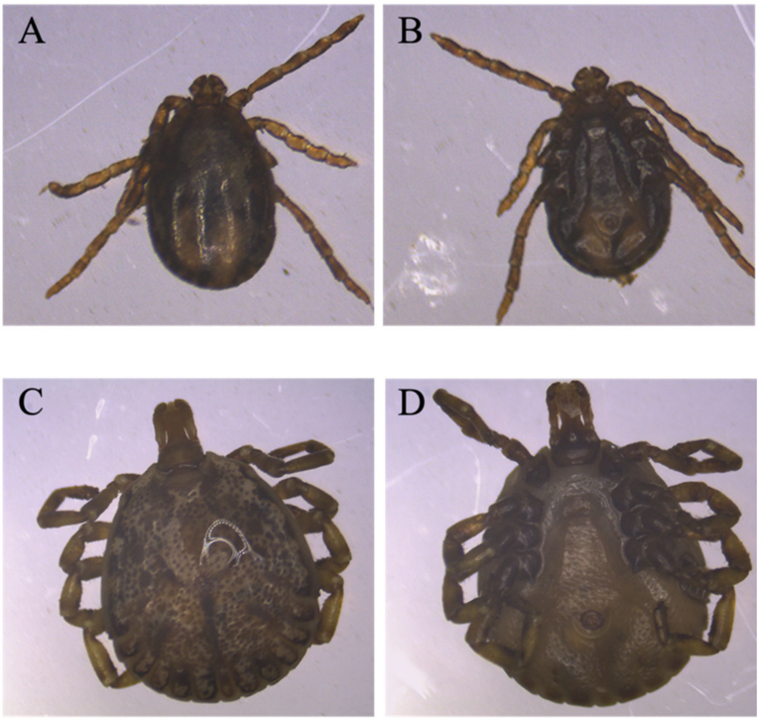

Figure 1 Dorsal and ventral morphological views of two tick genera collected from wild boars. (A) Dorsal view of *Haemaphysalis spp.*; (B) Ventral view of *Haemaphysalis spp.*;(C) Dorsal view of *Amblyomma spp.*; (D) Ventral view of *Amblyomma spp.*
