# Supplementary material for: Detection of Porcine Circovirus Type 3 in Free-Ranging Wild Boars and Ticks in Jiangsu Province, China
Source: Viruses. 2025 Jul 28;17(8):1049. doi: 10.3390/v17081049 (PMC12390564; doi:10.3390/v17081049)
Supplement: Supplementary file 1 [file viruses-17-01049-s001.zip › Table S1.pdf]

Table S1 Information about reference PCV3 genome sequences selected in the study.

Species	Country	Year	NCBI access number
Wild Boar	Brazil	2017	MT075517
		2017	MT075518
		2018	MT075519
	Spain	2005	MH579736
		2018	MH579747
	Korea	2020	MW168693
	Italy	2019	MN781189
	Germany	2018	MK820624
Domestic pig	China	2018	MK178322
	China	2017	MF589107
	China	2016	KY075993
	China	2016	MF069116
	China	2018	MK580465
	China	2017	MK580468
	China	2018	MK580466
	China	2017	MK580467
	China	2020	MZ449247
	China	2016	KY865242
	China	2020	MF677836
	China	2018	MH491016
	China	2016	KY075987
	China	2006	MG372488
	China	2006	MG372490
	USA	2016	NC031753
	USA	2015	KT869077
	Brazil	2018	MK645715
	Spain	2017	MT350542
	Korea	2017	MN698818
