# Supplementary material for: Detection of Porcine Circovirus Type 3 in Free-Ranging Wild Boars and Ticks in Jiangsu Province, China
Source: Viruses. 2025 Jul 28;17(8):1049. doi: 10.3390/v17081049 (PMC12390564; doi:10.3390/v17081049)
Supplement: Supplementary file 1 [file viruses-17-01049-s001.zip › Table S2 .pdf]

Table S2 Tick samples collection data, species and PCV3 detection results for each tick.

Wild boar ID	Tick species	Number of ticks	Positive for PCV3	Ct values for PCV3	Total
5	*Haemaphysalis longicornis*	3	0	-	5
	Amblyomma testudinarium	2	0		
6	*Haemaphysalis hystricis*	5	0	-	11
	Amblyomma testudinarium	6	0		
7	*Amblyomma testudinarium*	2	0	-	3
	Haemaphysalis longicornis	1	0		
8	*Amblyomma testudinarium*	2	2	33<br>35	2
9	*Amblyomma testudinarium*	18	3	34<br>33<br>34	18
19	*Haemaphysalis flava*	4	0	-	4
22	*Amblyomma testudinarium*	1	0	-	7
	Haemaphysalis flava	6	0		
34	*Amblyomma testudinarium*	6	0	-	6
45	*Amblyomma testudinarium*	11	1	36	11
47	*Haemaphysalis hystricis*	14	0	-	16
	Amblyomma testudinarium	2	0		

* “-” indicates PCV3-negative
